# The Impact of Matching Vaccine Strains and Post-SARS Public Health Efforts on Reducing Influenza-Associated Mortality among the Elderly

**DOI:** 10.1371/journal.pone.0011317

**Published:** 2010-06-25

**Authors:** Ta-Chien Chan, Chuhsing Kate Hsiao, Chang-Chun Lee, Po-Huang Chiang, Chuan-Liang Kao, Chung-Ming Liu, Chwan-Chuen King

**Affiliations:** 1 Institute of Epidemiology, College of Public Health, National Taiwan University, Taipei, Taiwan; 2 Division of Health Policy Research, Institute of Population Health Science, National Health Research Institutes, Zhunan, Taiwan; 3 Department of Public Health, College of Public Health, National Taiwan University, Taipei, Taiwan; 4 Department of Clinical Laboratory Science and Medical Biotechnology, College of Medicine, National Taiwan University, Taipei, Taiwan; 5 Global Change Research Center, National Taiwan University, Taipei, Taiwan; 6 Department of Atmospheric Sciences, College of Science, National Taiwan University, Taipei, Taiwan; Singapore Immunology Network, Singapore

## Abstract

Public health administrators do not have effective models to predict excess influenza-associated mortality and monitor viral changes associated with it. This study evaluated the effect of matching/mismatching vaccine strains, type/subtype pattern changes in Taiwan's influenza viruses, and the impact of post-SARS (severe acute respiratory syndrome) public health efforts on excess influenza-associated mortalities among the elderly. A negative binomial model was developed to estimate Taiwan's monthly influenza-associated mortality among the elderly. We calculated three winter and annual excess influenza-associated mortalities [pneumonia and influenza (P&I), respiratory and circulatory, and all-cause] from the 1999–2000 through the 2006–2007 influenza seasons. Obtaining influenza virus sequences from the months/years in which death from P&I was excessive, we investigated molecular variation in vaccine-mismatched influenza viruses by comparing hemagglutinin 1 (HA1) of the circulating and vaccine strains. We found that the higher the isolation rate of A (H3N2) and vaccine-mismatched influenza viruses, the greater the monthly P&I mortality. However, this significant positive association became negative for higher matching of A (H3N2) and public health efforts with post-SARS effect. Mean excess P&I mortality for winters was significantly higher before 2003 than after that year [mean ± S.D.: 1.44±1.35 vs. 0.35±1.13, p = 0.04]. Further analysis revealed that vaccine-matched circulating influenza A viruses were significantly associated with lower excess P&I mortality during post-SARS winters (i.e., 2005–2007) than during pre-SARS winters [0.03±0.06 vs. 1.57±1.27, p = 0.01]. Stratification of these vaccine-matching and post-SARS effect showed substantial trends toward lower elderly excess P&I mortalities in winters with either mismatching vaccines during the post-SARS period or matching vaccines during the pre-SARS period. Importantly, all three excess mortalities were at their highest in May, 2003, when inter-hospital nosocomial infections were peaking. Furthermore, vaccine-mismatched H3N2 viruses circulating in the years with high excess P&I mortality exhibited both a lower amino acid identity percentage of HA1 between vaccine and circulating strains and a higher numbers of variations at epitope B. Our model can help future decision makers to estimate excess P&I mortality effectively, select and test virus strains for antigenic variation, and evaluate public health strategy effectiveness.

## Introduction

Increased influenza vaccination coverage for the elderly, one of the highest risk groups for influenza-related deaths [1], has prevented influenza-related complications and deaths, based on 64 studies from 1964 to 2006 [2]. In Taiwan, elderly populations (aged ≥65 years) have received free influenza vaccination since 1998. Vaccine coverage rates have increased from 9.9% in 1998 to 49.1% in 2007. Despite similar expansions in coverage, pneumonia and influenza (P&I) mortality among the elderly have continued to rise in Italy [3] and the United States [4], [5]. Such findings contribute to the current international debate on the influenza vaccine's effectiveness in preventing elderly influenza-associated deaths. To examine this issue, we investigated the impact of potential vaccination mismatches with co-circulating viral strains of influenza virus types/subtypes, and public health efforts after the 2003 outbreak of SARS on vaccination effectiveness in subtropical regions like Taiwan.

Routine virological surveillance has been crucial for early detection of influenza viral changes [6]. Understanding epidemiological pattern changes of influenza in Taiwan, located geographically close to several past influenza pandemic epicenters in China and Southeast Asia, has larger implications for global virological surveillance. Taiwan's dominant circulating A(H3N2), A(H1N1), and B wild-type influenza virus strains appeared about one to two years earlier than selected vaccine strains recommended by World Health Organization (WHO) for the northern hemisphere, implying that Taiwan has the potential to play a key role in early pandemic and epidemic detection and control [7], [8].

The aims of this study were: (1) to evaluate the effectiveness of matching or mismatching influenza vaccine strains on influenza-associated mortality, (2) to assess whether public health improvements during the post-SARS period might have decreased elderly mortality, and (3) to investigate molecular variation among vaccine-mismatched influenza viruses that may be associated with increased excess influenza-associated mortality.

## Methods

### Data Sources and Definition of Influenza Seasons

Data was collected on Taiwan's annual and monthly influenza-associated mortality rates for the elderly population, monthly meteorological conditions (obtained from Taiwan's Central Weather Bureau), annual influenza vaccine strains (collected from the WHO) [9], dominant types/subtypes of influenza viruses for winter epidemic seasons, and monthly influenza isolation rates [compiled from the Centers for Disease Control in Taiwan (Taiwan-CDC)] for the 1999–2000 through 2006–2007 influenza seasons. Rates of both winter and annual influenza-associated excess mortality among the elderly were calculated. Winters periods were designated as December 1^st^ to February 28^th^ of the following year. Annual periods were marked as beginning on October 1^st^ and concluding on September 30th of the following year. The elderly population was calculated as the average of two mid-years' elderly population (acquired from the Census database of the Ministry of the Interior). Monthly isolation rates of influenza virus types/subtypes for each studied year were obtained from the Virological Surveillance Database of Contract Laboratories and compiled by Taiwan-CDC (Figure S1) [10]. Wild-type, dominant circulating influenza virus strains were also collected from the Contract Laboratories, as designated by Taiwan-CDC and the literature [8]. Comparisons between influenza vaccine strains and Taiwan's dominant influenza epidemic strains are summarized in Table S1.

To better evaluate influenza's disease burden, we used influenza-associated mortality rates that were calculated under broad definitions [4]. Using International Classification of Diseases, ninth revision (ICD-9) codes and clinical data obtained from Taiwan's Department of Health, mortality was divided into three categories: (1) pneumonia and influenza (P&I, ICD-9: 480–487), (2) respiratory and circulatory (R&C, ICD-9: 390–519), and (3) all-cause deaths (ICD-9: 000–999) with the exclusion of non-natural deaths.

### Data Analyses and Statistical Tests

We developed a negative binomial regression model and added two variables of vaccine match/mismatch and pre/post-SARS effect for multivariate analyses with a modification of a Thompson-like model [4], because of dispersed distributions of the three influenza-associated mortality rates (variance/mean >20). Explanatory variables for the above three outcome measures include monthly meteorological parameters (monthly means of temperature and humidity), annual periodic cycle (i.e., sine/cosine function of seasonal periodicity), monthly virus isolation rates for different subtypes/types of influenza viruses [A (H3N2) or A (H1N1) or B], matching status of different vaccine strains for each subtype/type in each of the studied years, post-SARS effect, and linear temporal monthly trends. Matching status for each subtype/type of influenza viruses was defined as the consistency between the nomenclature of the vaccine strain and the nomenclature of that season's dominant wild-type strain in Taiwan. If no wild-type subtype was isolated for a certain year, the status of the flu vaccine was thus coded as “matching” for that subtype/type and year. To assess whether the public health effort after the 2003 unique outbreak of SARS in Taiwan might also play a role in mortality, the indicator variable “post-SARS effect” was applied to the period beginning October 2003 (the first month of the 2003 influenza season) till after the conclusion of the outbreak in June 2003.

Model selection was based on Akaike's information criterion (AIC) and likelihood ratio test [11]. When modeling P&I mortality, both mean temperature and relative humidity were found to be without statistical significance and were thus excluded from the full model (Table 1). The model was implemented by SAS (version 9.1; SAS Institute Inc, Cary, NC). The final model for “influenza-associated deaths” was devised as follows:
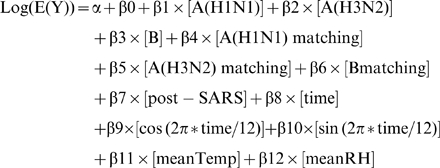



**Table 1:** Estimated coefficients (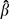), standard errors (SE) and p-values (p) of three fitted negative binomial models for influenza-associated deaths: (1) pneumonia and influenza (P&I), (2) respiratory and circulatory, and (3) all-cause in Taiwan, from October 1999 to September 2007, respectively.

	(1) P&I Deaths			(2) Respiratory and Circulatory Deaths			(3) All-Cause Deaths		
Parameters	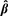	SE	p	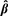	SE	p	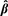	SE	p
Intercept, β_0_	−8.996	0.061	**<.0001** *	−5.326	0.214	**<.0001** *	−4.628	0.158	**<.0001** *
A/H1N1 Isolation Rate, β_1_	0.569	0.393	0.147	−0.183	0.165	0.268	−0.135	0.122	0.271
**A/H3N2 Isolation Rate, β_2_**	0.860	0.335	**0.010** *	0.326	0.139	**0.019** *	0.250	0.103	**0.015** *
Flu B Isolation Rate, β_3_	−0.133	0.208	0.522	−0.072	0.088	0.408	−0.025	0.065	0.701
A/H1N1 Vaccine Strain Matching, β_4_	0.192	0.111	0.085	−0.058	0.045	0.202	−0.028	0.034	0.401
**A/H3N2 Vaccine Strain Matching, β_5_**	−0.244	0.060	**<.0001** *	−0.002	0.025	0.923	−0.001	0.018	0.949
Flu B Vaccine Strain Matching, β_6_	−0.030	0.058	0.612	−0.050	0.024	**0.038** *	−0.027	0.018	0.131
**Post-SARS Effect, β_7_**	**−0.380**	**0.105**	**0.000** *	−0.067	0.043	0.116	−0.047	0.032	0.139
Linear Temporal Trends, β_8_	0.009	0.002	**<.0001** *	0.001	0.001	0.364	0.001	0.001	0.474
Cosine, β_9_	−0.081	0.020	**<.0001** *	0.033	0.025	0.183	0.056	0.018	**0.002** *
Sine, β_10_	0.087	0.021	**<.0001** *	−0.104	0.030	**0.001** *	−0.104	0.022	**<.0001** *
Mean Temperature, 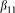	NA			−0.041	0.007	**<.0001** *	−0.035	0.005	**<.0001** *
Relative Humidity, 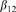	NA			−0.005	0.002	**0.017** *	−0.004	0.002	**0.011** *

*Statistically significant (p<0.05).

NA, Excluded by the Akaike Information Criterion (AIC) and likelihood ratio test.

α is an offset term equal to the log of elderly mid-year population size for each year.

**β_4_∼β_6_**, coded as “1” if the vaccine of that subtype/type had been matched and “0” for others.

**β_7_** (Post-SARS Effect), coded as “1” for the months after October, 2003 and “0” for others.

**β_8_**, coded 1 through 96 from October, 1999 to September, 2007. If no wild-type strain in a subtype was isolated for a certain year, the status of the flu vaccine was thus coded as “matching” for that subtype and year.

### Winter and annual influenza-associated excess elderly mortalities

To evaluate the impact of influenza vaccination and/or post-SARS effect on influenza-associated elderly mortalities, we listed both variables in Table 2. Excess mortality (95% confidence interval) was calculated for each winter and year. These excess deaths were assessed by calculating the difference between observed data and expected baselines that were derived from the negative binomial model (Figure S2). When we modeled vaccine-matching status and post-SARS effect, we coded data as “1's” or “0's” depending on the actual data for each year. “Post-SARS” was defined as all months after October 2003. After calculating residual deaths for each month, we replaced negative residuals (e.g. observed values less than the expected value) with zero and summed up excess deaths for each influenza season. Winter (or annual) excess mortality rates were calculated based on each winter's (or annual) total excess deaths divided by mid-year mean populations. In other words, we assessed temporal differences between each winter's monthly observed influenza-associated deaths and monthly expected value [obtained from our multivariate modeled deaths (baseline)], and then divided these monthly differences by the mid-year mean population for two years to calculate the actual excess mortality rate in each winter for statistical comparison among the three time periods (prior to SARS, during SARS, and post-SARS) (Table 2, Table S2). The mean values for each winter's excess mortality before and after SARS were evaluated by independent T-test.

**Table 2:** Annual and winter excess mortality rates of influenza-associated deaths (per 100,000) among the elderly (≧65 years).

Winter	Vaccine matching status & Post-SARS impact	Annual excess mortality		Winter excess mortality	
(By Years)		(95% CI)		(95% CI)	
**A. Pneumonia and Influenza Excess Mortality**					
1999–2000	Mismatched H1 & H3+without post-SARS	8.1	(3.5–14.3)	3.0	(2.0–4.0)
2000–01	Mismatched B+without post-SARS	6.9	(3.1–11.5)	6.2	(3.1–9.2)
2001–02	Mismatched B+without post-SARS	14.4	(6.5–25.5)	6.5	(2.8–10.9)
2002–03	Matched H3 & H1& B+without post-SARS	12.6	(6.1–21.8)	1.4	(0.0–3.6)
2003–04	Mismatched H3 & B+post-SARS	9.1	(3.6–20.8)	4.0	(1.9–7.1)
2004–05	Mismatched H3 & B+post-SARS	10.0	(5.7–17.6)	0.2	(0.0–2.6)
2005–06	Matched H3 & H1 & B+post-SARS	8.7	(4.8–20.9)	0.2	(0.0–2.7)
2006–07	Matched H3 & H1 & B+post-SARS	4.4	(1.9–14.5)	0.0	(0.0–3.5)
**B. Respiratory and Circulatory**					
1999–2000	Mismatched H1 & H3+without post-SARS	49.6	(21.4–76.9)	23.4	(8.1–38.1)
2000–01	Mismatched B+without post-SARS	21.2	(5.3–44.7)	16.5	(5.3–28.2)
2001–02	Mismatched B+without post-SARS	36.0	(15.8–67.6)	20.4	(9.0–35.2)
2002–03	Matched H3 & H1& B+without post-SARS	28.3	(15.9–60.4)	0.0	(0.0–8.7)
2003–04	Mismatched H3 & B+post-SARS	21.3	(6.0–51.1)	7.4	(1.6–20.1)
2004–05	Mismatched H3 & B+post-SARS	30.4	(6.6–64.0)	2.8	(0.0–10.8)
2005–06	Matched H3 & H1 & B+post-SARS	33.1	(13.7–65.5)	9.0	(2.5–20.1)
2006–07	Matched H3 & H1 & B+post-SARS	17.2	(8.7–50.9)	1.1	(0.0–13.9)
C. **All-Cause**					
1999–2000	Mismatched H1 & H3+without post-SARS	75.0	(23.0–133.7)	33.6	(6.0–60.6)
2000–01	Mismatched B+without post-SARS	43.6	(14.8–103.3)	29.5	(12.1–51.1)
2001–02	Mismatched B+without post-SARS	63.3	(24.8–114.5)	31.6	(11.3–51.8)
2002–03	Matched H3 & H1& B+without post-SARS	61.0	(31.9–118.7)	0.9	(0.0–18.2)
2003–04	Mismatched H3 & B+post-SARS	30.7	(5.6–96.8)	14.9	(0.0–44.2)
2004–05	Mismatched H3 & B+post-SARS	69.6	(27.5–135.7)	5.8	(0.0–16.7)
2005–06	Matched H3 & H1 & B+post-SARS	59.2	(24.8–123.7)	22.4	(7.2–43.2)
2006–07	Matched H3 & H1 & B+post-SARS	45.8	(17.0–119.7)	3.9	(0.0–31.2)

**#Annual:** from October to the following September.

**Winter:** from December to the following February.

### Phylogenetic Analysis of HA1 in Taiwanese H3N2 Viruses

To better understand why 2001–2002 exhibited the highest winter and annual excess P&I mortality rates, HA1 sequences of the 2002 epidemic influenza virus A (H3N2) Fujian strain (A/Fujian/411/2002 (H3N2)), vaccine strains (red squared symbol) from 1999 to 2007, and other Taiwanese H3N2 isolates (circular symbol) from 1996 to 2008 were gathered from the National Center for Biotechnology Information (NCBI) for genetic comparison. We then constructed a phylogenetic tree of HA1 amino acids among Taiwanese A (H3N2) virus strains using the Neighbor-Joining method. This tree was bootstrapped 1,000 times with Mega 4.0 software [12].

### Amino Acid Identity and Qualitative Analysis of Epitopes of HA1 of Influenza Vaccine-mismatched Circulating Versus Vaccine A (H3N2) Virus Strains

Percentages of amino acid sequence identity in the HA1 between dominant wild-type circulating and vaccine strains were calculated for vaccine-mismatched influenza A(H3N2) viruses isolated from months and years (1999–2000, 2003–2004, and 2004–2005) with high annual/monthly influenza-associated excess P&I mortality. The numbers and percentages of amino acid difference at specific epitope locations of HA1 that have been documented in literature [13] were further analyzed for vaccine-mismatched influenza A (H3N2) viruses isolated from these three years.

## Results

### Temporal Patterns of Three Influenza-Associated Mortality Rates

**Figure 1:**
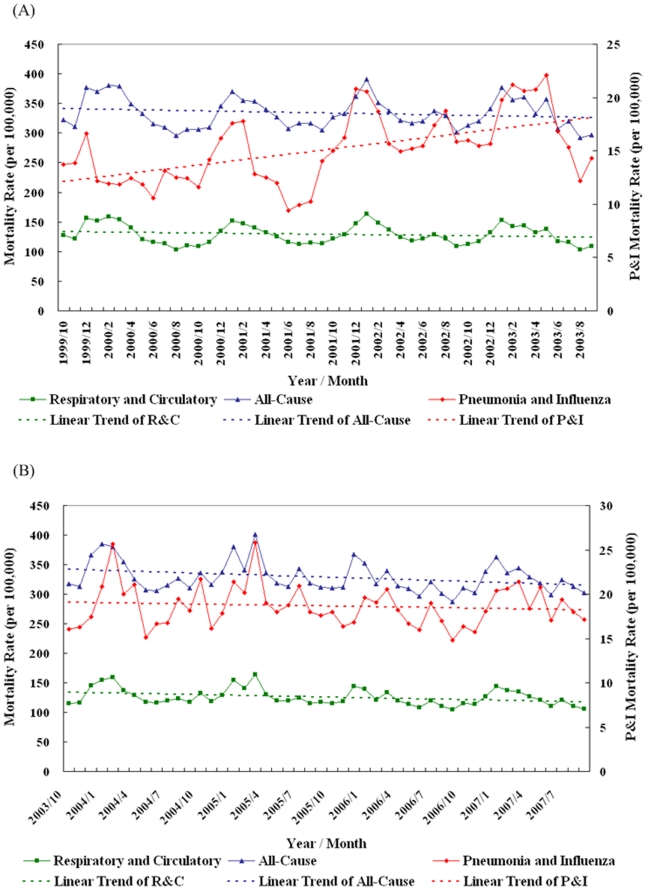
Monthly influenza-associated mortality rates for the 1999–2000 through 2006–2007 influenza seasons in Taiwan. **Left Y-axis** represents respiratory and circulatory mortality and all-cause (natural deaths) mortality. **Right Y-axis** represents pneumonia and influenza mortality. **Panel A** represents prior and during SARS period from October 1999 to September 2003. **Panel B** represents post-SARS period from October 2003 to September 2007. Three linear regression lines for the three corresponding influenza-associated mortality rates were drawn as dotted lines: (1) pneumonia and influenza mortality [red line], (2) respiratory and circulatory mortality [green line], and (3) all-cause (natural deaths) mortality [blue line].

Temporal patterns of influenza-associated mortality rates indicate that Taiwan's elderly P&I mortalities have been increasing since the beginning of the 2001–2002 influenza season despite significant increases in vaccine coverage (Figure S3A). As described in Figure 1, the mean P&I mortality rate, 12.97 per 100,000 prior to September 2001, significantly surged to 18.17 per 100,000 (p-value <0.001) after October 2001 (after the open travel policy with China commenced on January 1, 2001) [14]. These increasing patterns were consistent throughout the study period among the three oldest age groups (65–74, 75–84, and 85+) (Figure S3B). In addition to this temporal pattern, seasonal cycles (cosine and sine function in Table 1) were also found to be significant (p<0.01) for all three influenza-associated mortality rates (except for the cosine function for R&C). Furthermore, the corresponding coefficients in Table 1's model demonstrate the considerable reduction in elderly P&I mortality in years with either matching vaccines or post-SARS effect.

### Virological Surveillance and Mismatched Vaccine Strains

Temporal patterns of monthly isolation rates for human influenza viruses in Taiwan are displayed in Figure S1. During the study period, A (H3N2) and B were the dominant type/subtypes of influenza viruses. Influenza A (H1N1) was dominant in the 2001–2002 and 2005–2006 seasons, based on information from Taiwan-CDC and NCBI gene bank. Additionally, A (H3N2) was dominant during all summer flu seasons after the 2003 SARS outbreak.

Regarding to vaccine matching rate, A (H1N1) was the most frequently matched subtype (87.5%, 7/8) in eight epidemic seasons. A (H3N2) had the second highest (62.5%, 5/8) and B had the worst (50%, 4/8).

### Influenza-Associated Mortality Models

Using multivariate negative binomial regression models, we found that four significant variables – the monthly isolation rate of influenza A (H3N2), vaccine mismatch with A (H3N2), linear temporal trends, and sine function – were all positively correlated with annual P&I deaths (p<0.05, Table 1). In contrast, post-SARS effect and cosine function were negatively correlated with annual P&I mortality (p<0.05). Influenza A (H3N2)'s monthly isolation rate and sine function, as well as vaccine mismatch for influenza B, were positively correlated with R&C deaths (p<0.05). Mean temperature and relative humidity were both negatively correlated with R&C deaths (p<0.05). Values for observed and estimated deaths were proximate for all three influenza-associated mortality models (as illustrated in Figure S2).

### Excess Mortality, Post-SARS Effect, and Vaccine Match/Mismatch

#### 1. 2003 SARS Outbreak

During Taiwan's SARS epidemic (March–June 2003), excess P&I mortality rates (11.0 per 100,000) were 2–5 times higher than rates for the same months in prior years and about 8 times higher than rates for the four months preceding the outbreak (Table S2). In May 2003, when inter-hospital nosocomial infection was the most severe, the three influenza-associated excess mortality rates of P&I (22.09 per 100,000), R&C, and all-cause ranked the highest throughout the studied months. After June 2003, excess P&I mortalities in July and August declined dramatically to 0 per 100,000 (Figure S2).

#### 2. Pre- and Post-SARS Periods

The mean of excess influenza-associated mortalities of P&I and R&C for winters before 2003 was significantly higher than after 2003 (i.e. post-SARS period) [P&I excess mortality: mean ± standard deviation (S.D.): 1.44±1.35 vs. 0.35±1.13, p = 0.04; R&C excess mortality: 5.0/100,000 vs. 1.7/100,000, p = 0.04].

#### 3. Stratification Analysis

Totally, four of the eight studied years showed increased winter excess influenza-associated mortalities (≧3/100,000) plus one higher excess mortality in March, 2005 (5.28/100,000), and five of them had vaccine-mismatches. Of the latter five, three occurred in the pre-SARS period (1999–2000, 2000–2001, and 2001–2002) and the remaining two happened in the post-SARS period (2003–2004 and 2004–2005). To examine the winter seasons' excess mortality in those years with vaccine-matched versus vaccine-mismatched strains before and after SARS, we first reviewed the data on vaccine-matches or not during the pre-SARS years only (e.g. without effect of SARS). Then, we focused on vaccine-mismatches and compared it in pre- and post-SARS years (e.g. without effect of vaccine-matches).

In 2002–2003 winter (during the “pre-SARS” period), well vaccine-matched influenza strains resulted in a monthly mean of 0.45/100,000 excess P&I mortality. This was lower than vaccine-mismatched winters before 2002 [mean ± S.D.: 0.45/100,000±0.78 vs. 1.77/100,000±1.36, p = 0.2]. In other words, mismatched vaccine did show a trend in increased P&I mortalities in those years without post-SARS effect. In the 2005–2006 and 2006–2007 winters (during the “post-SARS period), well vaccine-matched influenza strains were also associated with lower excess P&I mortalities compared to the vaccine-mismatched winters during the “post-SARS” period [mean ± S.D.: 0.03/100,000±0.06 in 2005–07's winter months vs. 0.68/100,000±1.59 in those of vaccine-mismatched winters' months after 2003, p = 0.33], and the winter of 2002–2003 (i.e. 0.45/100,000, p = 0.4).

Comparing vaccine-mismatches in pre- and post-SARS winters, we found another trend: the excess P&I mortality mean in influenza vaccine-mismatched winters in pre-SARS was much higher than the mean in post-SARS winters (1.8±1.4 vs. 0.7±1.6, p = 0.2). In other words, post-SARS years showed a trend toward reduced influenza-associated mortality while vaccines were mismatched. Similar trends appeared that well influenza A (i.e. H1N1 and H3N2) vaccine-matches during post-SARS winters (i.e. 2005–2007) showed significantly lower excess P&I mortality than vaccine-matched influenza A viruses during pre-SARS winters [mean ± S.D.: 0.03±0.06 vs. 1.57±1.27, p = 0.01]. Furthermore, 2005–2006 and 2006–2007 had the two lowest P&I excess mortalities during the post-SARS period.

In summary, stratification of these two variables showed substantial trends toward lower excess P&I mortality during: (1) influenza vaccine-matched winters during the pre-SARS period (without post-SARS effect), and (2) influenza vaccine-mismatched winters in the post-SARS period.

### HA1 Amino Acid Identity Percentage, Phylogenetic Analysis and Epitope Variation of Taiwanese H3N2 Isolates versus Vaccine H3N2 Strains

To find whether the molecular-variations of influenza viruses might correlate with annual influenza-associated excess mortalities during 1999–2000, 2003–2004, and 2004–2005 (Table 2), we investigated the percentage of amino acid identity between circulating and vaccine strains, their phylogenetic relatedness, and amino acid differences at specific epitope locations in the HA1 of Taiwanese influenza A (H3N2) versus vaccine A (H3N2) strains. After examining the amino acid identity of vaccines and their corresponding circulated viral strains, we found that the mismatched A (H3N2) virus in 1999–2000 had a HA1 amino acid identity percentage of 96.4%. This was a higher identity percentage than in 2003–2004 (93.2%) but a lower identity percentage than in 2004–05 (97.1%) (Table 3). These quantitative differences correlate with the P&I excess mortality observed in 1999–2000's winter season (3.0/100,000), which was lower than in 2003–2004's winter season (4.0/100,000) but higher than in 2004–2005's winter season [0.2/100,000 (Table 2)]. The high winter and annual excess P&I mortalities in 2001–2002, before the 2003 SARS outbreak, can be explained in part by the 90.4% identity percentage of vaccine strain B/Sichuan/379/99 to the B/Hong Kong/330/2001 strain. This time period also began the emergence of the A (H3N2) Fujian strain in the region. After the 2003 SARS outbreak, winter excess mortality in 2003–2004 ranked the highest due to low identity (93.2%) of the vaccine strain [A/Moscow/10/99-like (H3N2)] to the existing A/Fujian/411/2002 (H3N2) virus. In March 2005, when excess influenza-associated P&I mortality peaked (5.28 per 100,000) during October 1^st^ 2004–September 30^th^ 2005, the isolated influenza B strains of Victoria lineage (B/Malaysia/2506/2004) [15] were shown to be mismatched (89.9% amino acid identity) with vaccine strains of Yamagata lineage (B/Shanghai/361/2002-like) [16], [17]. High excess mortality was therefore observed in March of that year rather than in the winter season during December 1^st^ 2004–February 28 2005.

**Table 3:** Amino acid sequence identities between vaccine strains and dominant wild-type strains of A (H3N2) in Taiwan from 1999 through 2007.

	Amino Acid Sequence Identity Percentage (%)			
	Vaccine Strains			
Dominant Strains	A/Sydney/5/97-like (H3N2)	A/Moscow/10/99-like (H3N2)	A/Fujian/411/2002-like (H3N2)	A/Wisconsin/67/05-like (H3N2)
**A/Sydney/05/97 (H3N2)**	0.996	0.96*	0.932	0.911
**A/Moscow/10/99 (H3N2)**	0.975	0.975	0.939	0.925
**A/Fujian/411/2002 (H3N2)**	0.935	0.932**	1	0.957
**A/California/7/2004 (H3N2)**	0.914	0.911	0.971***	0.971
**A/Wisconsin/67/05 (H3N2)**	0.911	0.903	0.964	0.985

*The vaccine was mismatched in the 1999–2000 season.

**A/Fujian/411/2002 was not a dominant strain during 2001–2002's season. The Vaccine was mismatched during the 2003–2004 season.

***The Vaccine was mismatched in the 2004–2005 season.

Phylogenetic analysis of amino acids in the HA1 of Taiwanese A (H3N2) viruses from 1996 to 2008 (Figure S4) displays A (H3N2) isolates obtained from the same year as clusters. The A (H3N2) Fujian strain, firstly isolated from China in August of 2002 and circulated dominantly in 2003 in Taiwan with notably high excess P&I mortality, was found to be phylogenetically distinct from other A(H3N2) strains.

To investigate whether more variation of HA1 epitopes between vaccines and dominant circulating strains might lead to higher excess mortality, we compared Taiwan's dominant circulating H3N2 strains with the vaccine H3N2 strains used during 1999–2000, 2003–2004, and 2004–2005 (Table S4, Figure S4). During the 1999–2000 flu season, A/Sydney/05/97(H3N2) and A/Moscow/10/99(H3N2) were the dominant circulating strains affecting Taiwan while A/Sydney/05/97-like was the vaccine strain and provided inadequate protection against A/Moscow/10/99. As shown in Table S3, five variations were identified at known epitopes [13], including two variations (Y137S, S142R) at epitope A [17% (2/12)], two (K160R, A196T) at epitope B [13% (2/16)], and one (R57Q) at epitope E [8% (1/13)]. No variations were found at epitopes C (0/8) or D (0/13) (Table S4). Most notably, we discovered one variation at the undefined epitope (position 3, I3L) previously identified by Shih et al. [13], and three new variations (positions of A138S, I194L, Y233H, Figure S5) without documentation in the literature. During the 2003–2004 flu season that followed the SARS outbreak, the dominant A/Fujian/411/2002 (H3N2) strain [mismatched with vaccine strain A/Moscow/10/99 (H3N2)] was phylogenetically distinct from other H3N2 strains and exhibited high epitope B variations (9/19, 47%). Surprisingly, nine of the 19 amino acid differences in HA occurred at epitope B. Fewer variations were observed at other epitopes [A (8%, 1/13), C (13%, 1/8), D (8%, 1/13), E (15%, 2/13)] (Table S4). During the 2004–2005 flu season, the dominant A/California/7/2004 (H3N2) strain did not match with the vaccine A/Fujian/411/2002 (H3N2) strain. Again, the major variations occurred mostly at epitope B (4/8, 50%). The remaining variations included one at epitope A [1/12 (8%) at K145N], two (V226I, S227P) at epitope D [2/13 (15%)], and one new variation at A138S that had appeared during the 1999–2000 flu season (Table S4). We also observed that the excess mortality in 2004–2005's winter was much lower than in 1999–2000. This can be supported by higher identity percentage between vaccine and circulating strains, and a lower number of variations at epitope B. In 2003–2004, there were more variations (9/19) at epitope B in circulating and vaccine-mismatched A (H3N2) strains than in the 2004–2005 influenza season. There was also a higher winter excess P&I mortality in 2003–2004 as compared to in 2004–2005.

## Discussion

This is the first study to analyze the impact of the dominant types/subtypes of influenza viruses, the matching status of influenza vaccine strains, and the 2003 SARS outbreak on three influenza-associated mortality rates among the elderly in Taiwan. The study is unprecedented in its molecular-level investigation of vaccine-mismatched influenza viruses associated with excess mortality. While the limitations of our data prevent us from drawing definitive conclusions about these potential factors, we did observe five associations that merit discussion. First, higher A (H3N2) subtype isolation rates were associated with increased influenza-associated mortality. Second, lower P&I mortality rates were observed when circulating strains of influenza viruses were vaccine-matched. Third, increased influenza P&I excess mortality was associated with vaccine-mismatched circulating influenza H3N2 and B viruses with fewer amino acid identities. Fourth, influenza disease burden after the 2003 SARS outbreak (i.e. with post-SARS effect) was significantly lower than before this SARS outbreak. Lastly, patterns of Taiwan's influenza types/subtypes became more diversified after 2001 when Mini links with China facilitated open travel exchanges [14]. Co-circulation of H3N2 subtype with H1N1 subtype or B type viruses resulted in higher P&I mortality than any subtype/type acting alone. Our observations suggest that improvements in public education and public health efforts (as a result of post-SARS effects and better vaccine matching) may have contributed to a reduction in P&I mortality. This trend would be further supported if mortality reductions persist in the presence of adequately sustained prevention measures. Our study suggests that the future deployment of epidemiological measures such as virological surveillance (obtaining more specimens), timely molecular analysis of viral isolates and their accompanying vaccine strains, and identification of vaccine-mismatched strains would support public health efforts to minimize complications and deaths. We recommend that public health resources be allocated to include both pharmaceutical [18] and non-pharmaceutical interventions [19] for minimizing elderly deaths whenever vaccine-mismatched H3N2 viruses are dominant. Daily syndromic surveillance data integrated with virological surveillance information and statistical methods for detecting abnormal signals and trends can provide timely information for identifying the occurrence of vaccine-mismatched or novel influenza viruses. These efforts can jump-start prevention at the initial phase of an epidemic, when there is a higher risk of human-to-human transmission (e.g., increased epidemic/pandemic potential of newly emerged influenza viruses). As outbreaks of emerging infectious diseases (EID) and novel influenza viruses continue to increase [20], we believe our results will help countries have not affected by SARS to evaluate the effectiveness of their preventive and/or control measures for reducing influenza disease burdens (including vaccination programs for the current influenza H1N1 pandemic in 2009–2010).

In addition to determining the effectiveness of vaccine strains in a given flu season, variant epitopes on the surface of the virus may also result in varying immunological responses. Certain epitope variants might be less effective at stimulating the development of B-cell humoral immunity or interfere with the ability of cytotoxic T-lymphocytes to recognize epitopes presented by HLA class I proteins on the surface of infected cells [21]. Analysis of data from pre-SARS winters—before the initiation of public health intervention efforts prompted by SARS—may provide more clues as to the impact of vaccine-mismatched influenza viruses on excess mortality. In addition, Taiwan's vaccine-mismatched influenza viruses appeared prior to the introduction of WHO's recommended vaccine strain [22]. Therefore, it is explainable why we observed higher excess P&I mortality during pre-SARS vaccine-mismatches than post-SARS. The mismatched A (H3N2) Fujian strain which appeared in 2002 and circulated for several months may have steadily increased the population's herd immunity leading up to 2004–2005. Lower influenza-associated excess mortality during the winter of 2004–2005, particularly in comparison to 1999–2000, may also be attributed to a higher identity percentage between vaccine strains and circulating strains, a lower number of variations at epitope B, the development of herd immunity, and the post-SARS effect. These two vaccine-mismatching examples suggest that both quantitative and qualitative variations of amino acids, as well as the locations and epitopes involved, are important considerations when monitoring vaccine-mismatched influenza viruses. Antigenic differences thus need to be identified efficiently by serological testing for isolated influenza viruses with high monthly/weekly excess P&I mortality [23]. Our results suggest that timely identification of vaccine-mismatched circulating influenza viruses and their antigenic variations is crucial for effective evidence-based public health planning and preparedness.

From January 2001–October 2001, documented vaccine effectiveness (VE) was 53% for preventing pneumonia deaths (when B was mismatched) and 44% for preventing all-cause deaths among the elderly in Taiwan [24]. Matched vaccines reached a VE as high as 80% for preventing influenza among healthy adults [25]. This VE declined to 50% when vaccines mismatched with circulating influenza viruses. We can surmise from past data that the VE for mismatched vaccines would be even lower for elderly populations because of their weakened immune responses [26]. The variation of VE across different countries may be attributed to different age distributions, variable influenza vaccination coverage rates [27], and variation in post-SARS effects. These variations may account for the higher overall P&I mortality and excess P&I mortality rates observed in Italy and U.S compared to Taiwan.

The 2003 SARS outbreak posed a significant challenge to Taiwan's health care system but also had the potentially beneficial effect of educating the public about the need for seeking health care earlier [28] and protective behaviors [29], [30]. Public education measures and behavioral changes prompted by the SARS outbreak may have contributed to the ensuing decline in excess P&I mortality in Taiwan and other SARS-affected countries/areas [29], [31]. Although our observation periods were not long, the evidence suggests that the SARS outbreak not only spurred behavioral change among Taiwanese citizens [32] but also prompted government officials to reform the infectious disease surveillance system [33] as well as policies for hospital management [34] and infection control [35] in Taiwan. A notable, quantifiable change that occurred following the SARS outbreak was the peaking of Taiwan's elderly influenza vaccine coverage rate at 68.4% in 2003–2004. Vaccination rates significantly rose from a mean of 31.8% before 2003–2004 to a mean of 55.3% during 2004–2007 (p = 0.08). These changes, in combination with increasing awareness of infectious diseases among physicians, may have contributed to the sustained post-SARS effect that we propose had a significant impact on Taiwan's elderly P&I mortality rates. Three studies in Taiwan [36], in Wuhan City of Hubei Province in mainland China [30] and in Hong Kong [31] support the claim that public health efforts were sustained after the SARS epidemic. Interestingly, influenza-associated mortalities in Taiwan, southern China and Hong Kong in the post-SARS period were all lower than in the pre-SARS period (personal communication in the March 15–19, 2010 MISMS Oceania Regional Influenza Meeting and Workshop in Melbourne, Australia). Moreover, many public health measures taken in response to SARS–including advising sick students to stay at home, teaching coughing etiquette, providing hand-cleaning facilities in front of elevators and at building entrances, closing classes if more than three influenza-like illnesses occur in one class, and vaccinating high-risk populations—all had been applied during the 2009 influenza H1N1 pandemic. The effectiveness of these public health efforts is supported by the lower total number of P&I mortalities observed during the 2009–2010 winter season (ending February 26, 2010) than in previous years [37]. In addition, we found that people had a greater understanding of health protection measures during the 2009–10 pandemic influenza in SARS-affected areas/countries such as Taiwan and Hong Kong [38]. This protective effect may have contributed to the lower numbers of P&I deaths in 2009–10 compared to seasonal influenza in 2008–2009 and to the reduction in total laboratory-confirmed pandemic influenza H1N1 deaths in Taiwan (42 deaths from July 1, 2009 to May 8, 2010)) and Hong Kong (80 deaths from July 17, 2009 to April 15, 2010) [39].

This study has five major limitations. First, Taiwan's monthly influenza virus isolation rates prior to 1999 were not comprehensive. Second, weekly and monthly matching statuses were not available. Third, the benefit of vaccination may be underestimated because older elderly populations are at higher risk of developing severe complications and deaths [40]. Fourth, the unknown temporality of vaccinations and the presence of many possible uncontrolled confounders (such as variations in age-specific attack rates, prior accumulated immunity, socioeconomic conditions, nutrition factors, public health efforts, and viral characteristics of individual influenza viruses including infectivity, pathogenicity, transmissibility and virulence) could not be fully accounted for in this retrospective ecological study. Causal effects cannot be determined with certainty from observational studies comparing these groups (e.g., vaccine-matched versus vaccine-mismatched groups or pre-SARS versus post-SARS groups in this study). Additionally, uncontrolled confounders at the individual level are a major limitation of an ecological study design. Fifth, accounting for epitope variations in A (H3N2) viruses will require more amino acid sequencing and serological data of the HA1 over the course of several years. Enhanced virological surveillance in Asia, where mostly past pandemic influenza viruses have originated [7], [41], is urgently needed because most new wild-type influenza virus strains have appeared much earlier in Taiwan and South-East Asia [42] than in WHO-recommended vaccine strains [8], [22]. Furthermore, viral changes and co-circulating subtypes/types have been documented in the later periods of influenza epidemics [43]. Antigenic distance between the vaccine and circulating strains can be best measured by serologically testing simultaneously for vaccine strains and 20–30 local influenza isolates obtained from various time intervals of the same year (in which excess influenza-associated mortality is identified). Unfortunately, we did not have enough monthly retrospective samples to incorporate serological results into the model during the study period. The major limitation of this study was a lack of long-term data that prevents us from drawing definitive conclusions. However, we have illustrated possible associations between the observed reduction in P&I mortality and vaccine match on the one hand, and the post-SARS effect on the other hand. Our model is sufficiently flexible to apply to different scenarios in various countries. For minimizing a country's/global influenza disease burden, further studies will be required to validate the interpretations of our results that we have suggested. International collaboration on an integrated clinical, epidemiological, and virological/serological influenza surveillance system will be necessary to monitor for potential increases in clinical severity as well as viral sequence and antigenic changes in any parts of the world.

This study points to a number of possible directions for improving influenza vaccination policy and provides a means for public health officials to monitor for possible occurrences of vaccine-mismatched influenza viruses at the population level. Moreover, our study attempts to lay a foundation for a molecular explanation of influenza-associated deaths that integrates macro-level mortality data with micro-level amino acid sequence identity percentage. We hope that our findings can prompt the discovery of better and more effective mechanisms for selecting strains for future serological testing. In the future, we hope to collect more data domestically and internationally in order to reevaluate and refine our recommendations. Public health professionals in SARS-affected countries can also examine the post-SARS impact and vaccine-mismatched effect using data sets from their countries. A concerted effort to obtain more evidence can bring the international community closer to devising more effective guidelines for the global control of next pandemic influenza. Future research efforts should include: (1) weekly/daily monitoring of influenza viral sequences, antigenic changes of the HA [44], and excess influenza-associated mortality; (2) an evaluation of vaccine efficacy through measurement of antigenic distances [45], [46], and B- and T-cell epitopes [47]; and (3) improvements in influenza vaccines through enhancement of innate immunity [48], [49], [50].

## Supporting Information

Figure S1 Monthly isolation rates of human influenza viruses [A (H1N1), A (H3N2), and B] in Taiwan from October 1999 to September 2007.
(0.04 MB DOC)

Figure S2 Observed and estimated influenza-associated deaths in Taiwan from October 1999 to September 2007.
(0.15 MB DOC)

Figure S3 (A). Temporal trend in influenza vaccine coverage rates and elderly pneumonia and influenza mortality (crude versus age-adjusted mortality rates) in Taiwan, from 1998–1999 to 2006–2007 influenza seasons. (B). Pneumonia and influenza mortality among three elderly age groups in Taiwan, from 1998–1999 to 2006–2007 influenza seasons.
(0.03 MB DOC)

Figure S4 Phylogenetic analysis of amino acid sequences of HA1 proteins. Phylogenetic analysis of amino acid sequences of HA1 proteins in 64 Taiwanese human H3N2 viruses isolated from 1996 to 2008 and the three influenza vaccine virus strains recommended by WHO [A/Sydney/5/1997 (H3N2), A/Moscow/10/1999 (H3N2), and A/Fujian/411/2002 (H3N2)].
(0.14 MB DOC)

Figure S5 The 3D structure of the three newly undefined epitopes of human influenza A (H3N2) viruses during the three vaccine-mismatched influenza seasons in Taiwan, 1999–2007. Epitopes of A–E and newly undefined epitope regions were marked with different colors [Epitope A in red, Epitope B in yellow, Epitope C in purple, Epitope D in light blue, Epitope E in light brown, Old Undefined Epitope (documented in literature) in orange, Newly Undefined Epitope that we identified from this study in white shown by pink arrow].
(0.35 MB DOC)

Table S1 Yearly comparisons between vaccine strains and circulating wild-type dominant strains of human influenza A/H3N2 and A/H1N1 viruses isolated in Taiwan from the 1999–2000 to 2006–2007 epidemic seasons.
(0.04 MB DOC)

Table S2 Comparison between elderly excess pneumonia & influenza (P&I) deaths and mortality rates in Taiwan in winter and summer influenza Seasons from 1999–2000 to 2006–2007.
(0.04 MB DOC)

Table S3 Number of amino acid variations at A, B, C, D, E and Old/New Undefined Epitopes between co-/circulating and vaccine strains of human influenza A (H3N2) viruses in the 3 H3N2 vaccine-mismatched years in Taiwan.
(0.03 MB DOC)

Table S4 Amino acid variants at the specific sites that literature documented and Old/New Undefined Epitopes between vaccine and dominant circulating strains of A (H3N2) in Taiwan in the influenza seasons of 1999–2000, 2003–04 and 2004–05.
(0.04 MB DOC)
