# Supplementary material for: The Impact of Matching Vaccine Strains and Post-SARS Public Health Efforts on Reducing Influenza-Associated Mortality among the Elderly
Source: PLoS One. 2010 Jun 25;5(6):e11317. doi: 10.1371/journal.pone.0011317 (PMC2892467; doi:10.1371/journal.pone.0011317)
Supplement: Figure S1 — Monthly isolation rates of human influenza viruses [A (H1N1), A (H3N2), and B] in Taiwan from October 1999 to September 2007. (0.04 MB DOC) [file pone.0011317.s001.doc]

**Figure S1. Monthly Isolation Rates of Human Influenza Viruses [A (H1N1), A (H3N2), and B] in Taiwan from October 1999 to September 2007**

**^1^Data obtained from Taiwan-CDC**

**^2^During the 1999-2000 influenza season, the influenza A subtype was largely untyped in the database**
