# Supplementary material for: The Impact of Matching Vaccine Strains and Post-SARS Public Health Efforts on Reducing Influenza-Associated Mortality among the Elderly
Source: PLoS One. 2010 Jun 25;5(6):e11317. doi: 10.1371/journal.pone.0011317 (PMC2892467; doi:10.1371/journal.pone.0011317)
Supplement: Figure S2 — Observed and estimated influenza-associated deaths in Taiwan from October 1999 to September 2007. (0.15 MB DOC) [file pone.0011317.s002.doc]

**Figure S2. Observed and Estimated Influenza-Associated Deaths in Taiwan from October 1999 to September 2007**

**(1)Pneumonia and Influenza (P&I) Deaths**

**
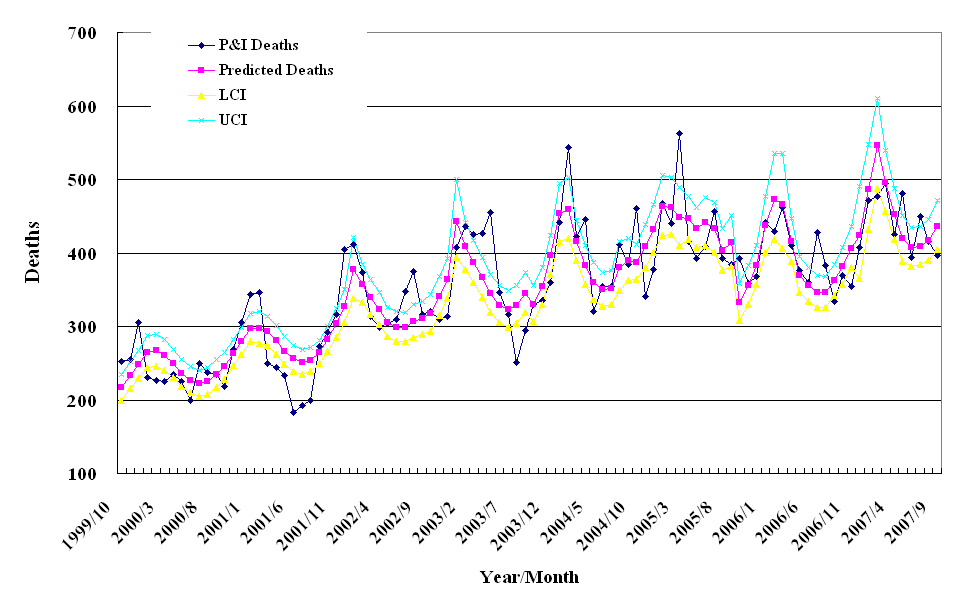
**

**(2)Respiratory and Circulatory (R&C) Deaths**


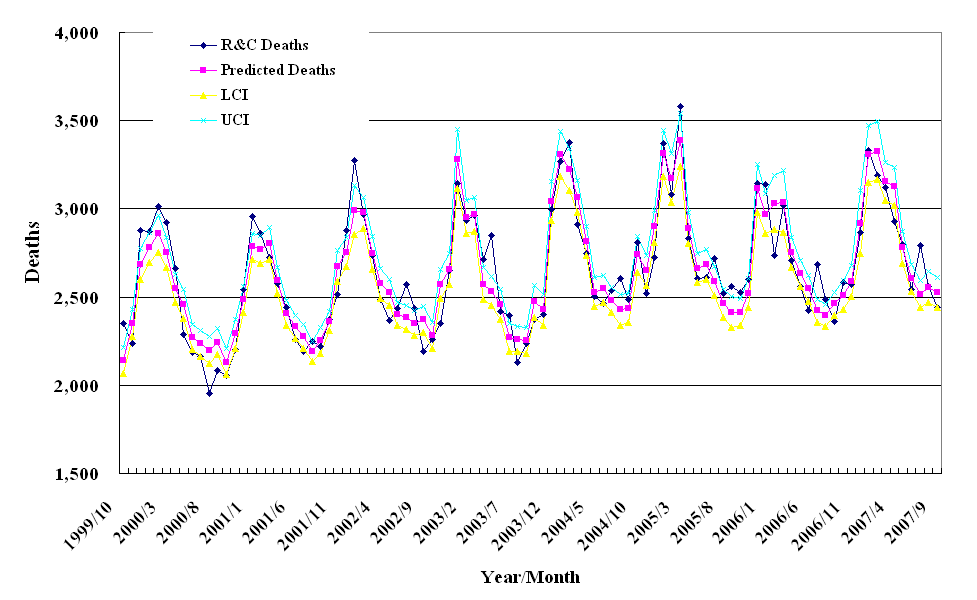


**(3) All-Cause Deaths**

**
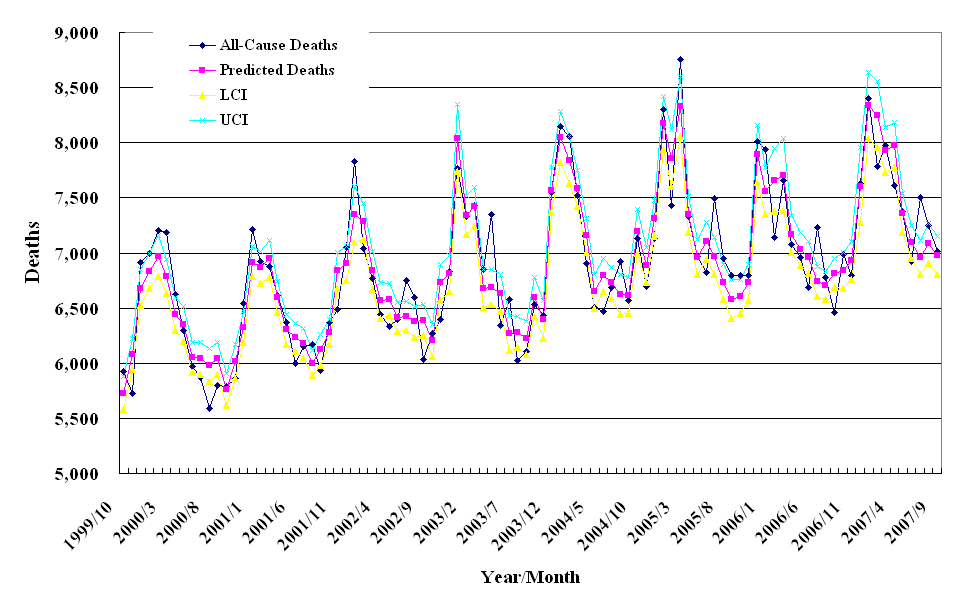
**

**LCI: Lower limit of 95% confidence interval of that estimate**

**UCI: Upper limit of 95% confidence interval of that estimate**
