# Supplementary material for: The Impact of Matching Vaccine Strains and Post-SARS Public Health Efforts on Reducing Influenza-Associated Mortality among the Elderly
Source: PLoS One. 2010 Jun 25;5(6):e11317. doi: 10.1371/journal.pone.0011317 (PMC2892467; doi:10.1371/journal.pone.0011317)
Supplement: Figure S3 — (A). Temporal trend in influenza vaccine coverage rates and elderly pneumonia and influenza mortality (crude versus age-adjusted mortality rates) in Taiwan, from 1998–1999 to 2006–2007 influenza seasons. (B). Pneumonia and influenza mortality among three elderly age groups in Taiwan, from 1998–1999 to 2006–2007 influenza seasons. (0.03 MB DOC) [file pone.0011317.s003.doc]

**Figure S3. (A). Temporal Trend in Influenza Vaccine Coverage Rates and Elderly Pneumonia and Influenza Mortality (Crude versus Age-adjusted Mortality Rates) in Taiwan, from 1998-1999 to 2006-2007 Influenza Seasons**

**Figure S3. (B). Pneumonia and Influenza Mortality in Taiwan among Three Elderly Age Groups in Taiwan, from 1998-1999 to 2006-2007 Influenza Seasons**

***Correlation between populations aged 65-74 and 75-84 was 0.71 (p=0.03).**

**Correlation between populations aged 65-74 and 85+ was 0.69 (p=0.04).**
