# Supplementary material for: The Impact of Matching Vaccine Strains and Post-SARS Public Health Efforts on Reducing Influenza-Associated Mortality among the Elderly
Source: PLoS One. 2010 Jun 25;5(6):e11317. doi: 10.1371/journal.pone.0011317 (PMC2892467; doi:10.1371/journal.pone.0011317)
Supplement: Figure S4 — Phylogenetic analysis of amino acid sequences of HA1 proteins. Phylogenetic analysis of amino acid sequences of HA1 proteins in 64 Taiwanese human H3N2 viruses isolated from 1996 to 2008 and the three influenza vaccine virus strains recommended by WHO [A/Sydney/5/1997 (H3N2), A/Moscow/10/1999 (H3N2), and A/Fujian/411/2002 (H3N2)]. (0.14 MB DOC) [file pone.0011317.s004.doc]

**Figure S4. Phylogenetic Analysis of Amino Acid Sequences of HA1 Proteins in 64 Taiwanese Human H3N2 Viruses Isolated from 1996 to 2008 and the Three Influenza Vaccine Virus Strains Recommended by WHO [A/Sydney/5/1997 (H3N2), A/Moscow/10/1999 (H3N2), and A/Fujian/411/2002 (H3N2)]**

**Legends:**


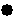
Taiwanese H3N2 viruses, 1996;
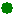
Taiwanese H3N2 viruses, 1997;
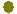
 Taiwanese H3N2 viruses, 1998;
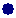
Taiwanese H3N2 viruses, 1999;
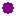
Taiwanese H3N2 viruses, 2000;
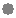
Taiwanese H3N2 viruses, 2002;
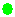
Taiwanese H3N2 viruses, 2003;
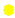
 Taiwanese H3N2 viruses, 2004;
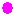
 Taiwanese H3N2 viruses, 2005;
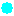
Taiwanese H3N2 viruses, 2006;
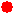
Taiwanese H3N2 viruses, 2007;
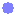
Taiwanese H3N2 viruses, 2008;
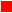
H3N2 vaccine strains used in northern hemisphere recommended by WHO from 1999-2000 influenza season through 2006-2007 influenza season.

**
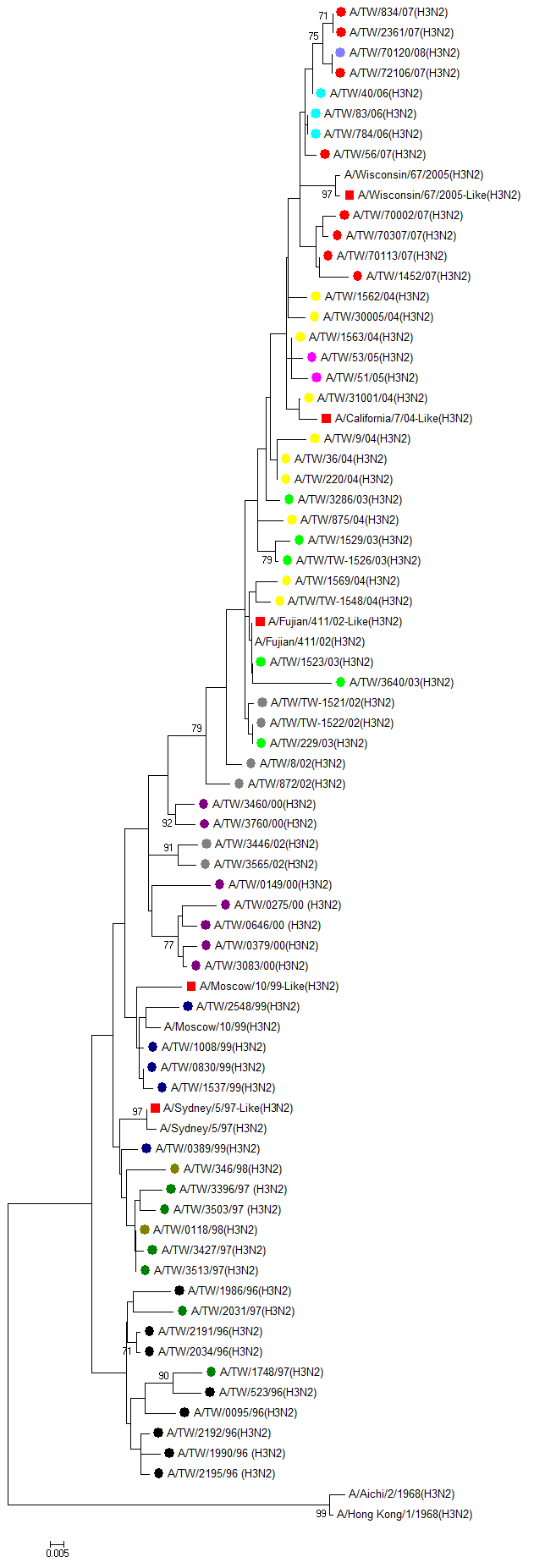
**
