# Supplementary material for: The Impact of Matching Vaccine Strains and Post-SARS Public Health Efforts on Reducing Influenza-Associated Mortality among the Elderly
Source: PLoS One. 2010 Jun 25;5(6):e11317. doi: 10.1371/journal.pone.0011317 (PMC2892467; doi:10.1371/journal.pone.0011317)
Supplement: Figure S5 — The 3D structure of the three newly undefined epitopes of human influenza A (H3N2) viruses during the three vaccine-mismatched influenza seasons in Taiwan, 1999–2007. Epitopes of A–E and newly undefined epitope regions were marked with different colors [Epitope A in red, Epitope B in yellow, Epitope C in purple, Epitope D in light blue, Epitope E in light brown, Old Undefined Epitope (documented in literature) in orange, Newly Undefined Epitope that we identified from this study in white shown by pink arrow]. (0.35 MB DOC) [file pone.0011317.s005.doc]

**Figure S5. The 3D Structure of the three Newly Undefined Epitopes of Human Influenza A (H3N2) Viruses during the three Vaccine-mismatched Influenza Seasons in Taiwan, 1999 - 2007**

**Legends:**

Epitopes of A-E and newly undefined epitope regions were marked with different colors [Epitope A in red, Epitope B in yellow, Epitope C in purple, Epitope D in light blue, Epitope E in light brown, Old Undefined Epitope (documented in literature) in orange**,** Newly Undefined Epitope that we identified from this study in white are shown by pink arrow].

**Figure (A):** The rear view of the 3D structure of HA of human A (H3N2) virus.

**Figure (B), (C), (D):** The three Newly Undefined Epitopes of A (H3N2) viruses that we identified from this study during the three Vaccine-mismatched Influenza seasons (1999-2000, 2003-2004, and 2004-2005) in Taiwan

**
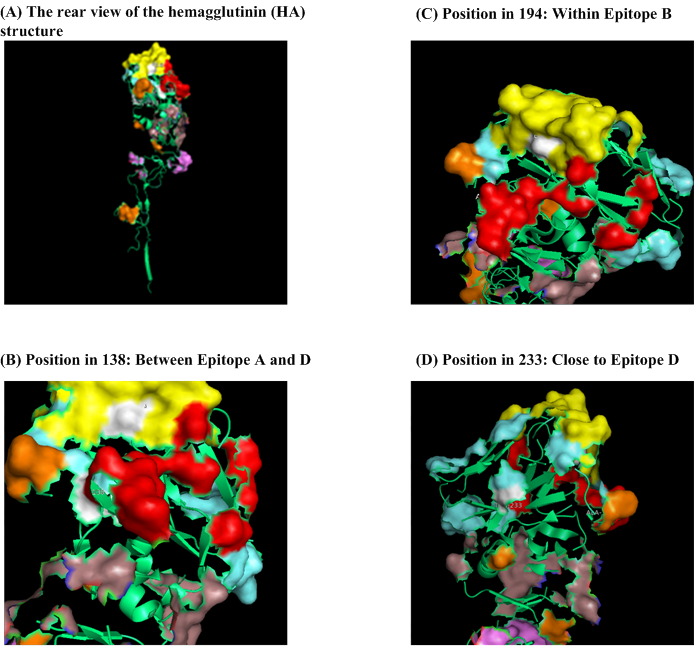
**
