# Supplementary material for: The Impact of Matching Vaccine Strains and Post-SARS Public Health Efforts on Reducing Influenza-Associated Mortality among the Elderly
Source: PLoS One. 2010 Jun 25;5(6):e11317. doi: 10.1371/journal.pone.0011317 (PMC2892467; doi:10.1371/journal.pone.0011317)
Supplement: Table S1 — Yearly comparisons between vaccine strains and circulating wild-type dominant strains of human influenza A/H3N2 and A/H1N1 viruses isolated in Taiwan from the 1999–2000 to 2006–2007 epidemic seasons. (0.04 MB DOC) [file pone.0011317.s006.doc]

**Table S1. Yearly Comparisons between Vaccine Strains and Circulating**

**Wild-type Dominant Strains of Human Influenza A/H3N2 and A/H1N1 Viruses**

**Isolated in Taiwan from** the 1999-2000 to 2006-2007 Epidemic Seasons

|  | **Influenza H1N1** | | |  | **Influenza H3N2** | | |  | **Influenza B** | | |
| --- | --- | --- | --- | --- | --- | --- | --- | --- | --- | --- | --- |
| **Year** | **Vaccine Strains*** | **Taiwan Isolates**** | **Match** |  | **Vaccine Strains*** | **Taiwan Isolates**** | **Match** |  | **Vaccine Strains*** | **Taiwan isolates**** | **Match** |
| **1999-2000** | Beijing/265/95-like | New Caledonia/20/99427/88 + Beijing/262/95 | **NO** |  | Sydney/05/97-like | Sydney/05/97 + **Moscow/10/99** | **NO** |  | Shangdong/07/97-like or Beijing/184/93-  like (Yamagata lineage) | Shangdong/07/97 + Beijing184/93 | YES |
| **2000-2001** | New Caledonia/20/99 -like | New Caledonia/20/99 | YES |  | Moscow/10/99 -like | Moscow/10/99 | YES |  | Beijing/184/93-like (Yamagata lineage) | Beijing184/93 + Sichuan/379/99 | **NO** |
| **2001-2002** | New Caledonia/20/99 -like | New Caledonia/20/99 | YES |  | Moscow/10/99 -like | Moscow/10/99 | YES |  | Sichuan/379/99-like (Yamagata) | Hong Kong/330/2001 | **NO** |
| **2002-2003** | New Caledonia/20/99 -like | New Caledonia/20/99 | YES |  | Moscow/10/99 -like | Moscow/10/99 | YES |  | Hong Kong/330/2001-like (Victoria lineage) | Hong Kong/330/2001 | YES |
| **2003-2004** | New Caledonia/20/99 -like | New Caledonia/20/99 | YES |  | Moscow/10/99 -like | **Fujian/411/2002** | **NO** |  | Hong Kong/330/2001-like (Victoria lineage) | Sichuan/379/99 | **NO** |
| **2004-2005** | New Caledonia/20/99 -like | NA | YES |  | Fujian/411/2002 -like | **California/7/2004** | **NO** |  | Shanghai/361/2002-like (Yamagata lineage) | Shanghai/361/2002 + Malaysia/2506/2004 | **NO** |
| **2005-2006** | New Caledonia/20/99 -like | New Caledonia/20/99 | YES |  | California/7/2004 -like | NA | YES |  | Shanghai/361/2002-like (Yamagata lineage) | NA | YES |
| **2006-2007** | New Caledonia/20/99 -like | NA | YES |  | Wisconsin/67/2005-like | Wisconsin/67/2005 | YES |  | Malaysia/2506/2004-like (Victoria lineage) | Malaysia/2506/2004 | YES |

***:** Vaccine strains recommended by the WHO ****:** Taiwan’s dominant strain NA= Not Available

Matching =YES indicates that the human influenza vaccine strains, recommended for the northern hemisphere by the WHO, were the same as the circulating dominant human influenza wild-type viruses isolated from that season in Taiwan
