# Supplementary material for: The Impact of Matching Vaccine Strains and Post-SARS Public Health Efforts on Reducing Influenza-Associated Mortality among the Elderly
Source: PLoS One. 2010 Jun 25;5(6):e11317. doi: 10.1371/journal.pone.0011317 (PMC2892467; doi:10.1371/journal.pone.0011317)
Supplement: Table S2 — Comparison between elderly excess pneumonia & influenza (P&I) deaths and mortality rates in Taiwan in winter and summer influenza Seasons from 1999–2000 to 2006–2007. (0.04 MB DOC) [file pone.0011317.s007.doc]

**Table S2.** **Comparison between Elderly Excess Pneumonia & Influenza (P&I)**

**Deaths and Mortality Rates in Taiwan in Winter and Summer Influenza Seasons from the 1999-2000 to 2006-2007 Influenza Seasons**

| **Epidemic Seasons** | **Months** | **Excess P&I Deaths** | **Excess P&I Mortality** |
| --- | --- | --- | --- |
|  |  |  | **(Per 100,000 Population)** |
| **1999-2000** | Nov. – Feb.**^1^** | 79.01 | 4.23 |
| **2000-2001** | Nov. – Feb.**^1^** | 125.66 | 6.54 |
| **2001-2002** | Nov - Feb | 141.47 | 7.16 |
| **2002-2003*** | Nov - Feb | 27.82 | 1.37 |
| **2003-2004** | Nov - Feb | 83.2 | 3.98 |
| **2004-2005** | Nov - Feb | 3.77 | 0.18 |
| **2005-2006** | Nov - Feb | 6.37 | 0.29 |
| **2006-2007** | Nov - Feb | 0 | 0 |
| **1999-2000** | Mar. – June**^2^** | 0 | 0 |
| **2000-2001** | Mar. - June**^2^** | 0 | 0 |
| **2001-2002** | Mar - June | 11.1 | 0.6 |
| **2002-2003*** | Mar - June | **226.4** | **11** |
| **2003-2004** | Mar - June | 74.9 | 3.5 |
| **2004-2005** | Mar - June | 115.3 | 5.3 |
| **2005-2006** | Mar - June | 9.5 | 0.4 |
| **2006-2007** | Mar - June | 61.9 | 2.7 |

*** The 2003 SARS epidemic in Taiwan**

**^1^** Winter Influenza Season

**^2^** Summer Influenza Season
