# Supplementary material for: The Impact of Matching Vaccine Strains and Post-SARS Public Health Efforts on Reducing Influenza-Associated Mortality among the Elderly
Source: PLoS One. 2010 Jun 25;5(6):e11317. doi: 10.1371/journal.pone.0011317 (PMC2892467; doi:10.1371/journal.pone.0011317)
Supplement: Table S3 — Number of amino acid variations at A, B, C, D, E and Old/New Undefined Epitopes between co-/circulating and vaccine strains of human influenza A (H3N2) viruses in the 3 H3N2 vaccine-mismatched years in Taiwan. (0.03 MB DOC) [file pone.0011317.s008.doc]

**Table S3. Number of Amino Acid Variations at A, B, C, D, E and Old/New Undefined Epitopes between Co-/circulating and Vaccine Strains of Human Influenza A (H3N2) Viruses in the 3 H3N2 Vaccine-mismatching Years in Taiwan**

| **Epitopes** | **# of Amino Acid Variations of the 5 Known and 4 Old/New Undefined Epitopes of Taiwanese A(H3N2) Viruses in the Following Three H3N2 Vaccine-mismatching Years.** | | |
| --- | --- | --- | --- |
|  | **1999-2000** | **2003-2004** | **2004-2005** |
| **Old- Undefined^1^** | 1 | 4 | 0 |
| **Newly Undefined^2^** | 3 | 1 | 1 |
| **A** | 2 | 1 | 1 |
| **B** | 2 | 9 | 4 |
| **C** | 0 | 1 | 0 |
| **D** | 0 | 1 | 2 |
| **E** | 1 | 2 | 0 |
| **Total** | 9 | 19 | 8 |

1. Described in Shih et al., PNAS., 104(15):6283-8, 2007.

2. Found in this study.
