# Supplementary material for: The Impact of Matching Vaccine Strains and Post-SARS Public Health Efforts on Reducing Influenza-Associated Mortality among the Elderly
Source: PLoS One. 2010 Jun 25;5(6):e11317. doi: 10.1371/journal.pone.0011317 (PMC2892467; doi:10.1371/journal.pone.0011317)
Supplement: Table S4 — Amino acid variants at the specific sites that literature documented and Old/New Undefined Epitopes between vaccine and dominant circulating strains of A (H3N2) in Taiwan in the influenza seasons of 1999–2000, 2003–04 and 2004–05. (0.04 MB DOC) [file pone.0011317.s009.doc]

**Table S4. Amino Acid Variants at the Specific Sites that Literature Documented and Old/New Undefined Epitopes between Vaccine**

**and Dominant Circulating Strains of A (H3N2) in Taiwan in the Influenza Seasons of 1999-2000, 2003-04 and 2004-05**

| **A (H3N2) Strains** | **Amino Acids at the Following Specific Positions (A, B, C, D, E, Old/New Undefined Epitopes)** | | | | | | | | |  |
| --- | --- | --- | --- | --- | --- | --- | --- | --- | --- | --- |
| **Year 1999-2000** | 3 (Old) | 57(E) | 137(A) | **138(New)** | 142(A) | 160(B) | **194(New)** | 196(B) | **233(New)** |  |
| Sydney/05/97-like* | I | R | Y | A | S | K | I | A | Y |  |
| Sydney/05/97 # | I | R | Y | A | S | K | I | A | H |  |
| Moscow/10/99 # | L | Q | S | S | R | R | L | T | Y |  |
| **Year 2003-2004** | 160(B) | 172(D) | 186(B) | 190(B) | 192(B) | **194(New)** | 196(B) | 202(Old) | 222 (Old) | 225(Old) |
| Moscow/10/99-like* | R | D | S | V | T | V | V | V | W | G |
| Fujian/411/2002 # | K | E | G | D | I | L | A | I | R | D |
| **Year 2004-2005** | **138(New)** | 145(A) | 159(B) | 188(B) | 189(B) | 196(B) | 226(D) | 227(D) |  |  |
| Fujian/411/2002-like* | A | K | Y | D | S | A | V | S |  |  |
| California/7/2004 # | S | N | F | N | N | T | I | P |  |  |

* H3N2 vaccine strains in those years # Dominant circulating strains in those years.

**Old Undefined Epitopes:** Documented in Shih et al. (2007).

**New Undefined Epitopes:** Identified from this study (shown by yellow background).
